# Reassessing high-dose ranibizumab in the era of durable anti-VEGF therapy

**DOI:** 10.3389/fopht.2026.1802903

**Published:** 2026-04-01

**Authors:** Cheng-Kuo Cheng, Cheng-Jui Tsai, Chyi-Huey Bai

**Affiliations:** 1 Department of Ophthalmology, Shin Kong Wu Ho-Su Memorial Hospital, Taipei, Taiwan; 2 School of Medicine, Fu-Jen Catholic University, New Taipei, Taiwan; 3 School of Medicine, National Taiwan University, Taipei, Taiwan; 4 Department of General Medicine, Shin Kong Wu Ho-Su Memorial Hospital, Taipei, Taiwan; 5 Department of Public Health, School of Medicine, Taipei Medical University, Taipei, Taiwan; 6 School of Public Health, Taipei Medical University, Taipei, Taiwan

**Keywords:** anti-vascular endothelial growth factor, dose escalation, injection frequency, neovascular age-related macular degeneration, treatment burden

## 1 Introduction

The management of neovascular age-related macular degeneration (nAMD) has evolved significantly, with a growing emphasis on treatment durability alongside visual efficacy. Recent approvals of high-dose aflibercept (8.0 mg) and faricimab, reflect a clinical shift toward extending treatment intervals to alleviate the injection burden on patients and healthcare systems (1–3). In light of this paradigm shift, the historical data regarding high-dose ranibizumab (2.0 mg) merit re-examination through the viewpoints of treatment burden.

The Phase 3 HARBOR trial compared 0.5 mg and 2.0 mg ranibizumab, concluding that the 2.0 mg dose did not demonstrate a clinically meaningful difference in efficacy or durability (4, 5). Consequently, the 0.5 mg dose remained the standard of care. However, the trial reports also included data on injection frequency in the pro re nata (PRN) arms. While numerical differences were observed, these were not the primary focus of the original statistical analysis (4, 5).

This article suggests that a retrospective statistical evaluation of the HARBOR data offers valuable insights into the relationship between dose escalation and injection frequency. High-dose ranibizumab (2.0 mg) appears to reduce injection frequency compared to the standard dose (0.5 mg), consistent with the PULSAR and PHOTON trials (1, 3).

## 2 The statistical oversight: a quantitative reassessment

The original HARBOR reports presented the mean number of injections in the PRN arms but did not perform formal hypothesis testing to compare injection frequencies between doses (4, 5). To understand the true value of the 2.0 mg dose, we revisited this summary data using a Welch’s t-test, which allows for robust comparison of means from independent samples with potentially unequal variances. Significance was defined as a two-sided p-value < 0.05, and results are presented with the corresponding p-values and 95% confidence intervals (CI). Statistical analyses were performed using R version 4.4.3 with the Basic Statistics and Data Analysis package.

The retrospective analysis reveals a significant reduction in treatment burden that was previously described only as a numerical trend. Injection counts are reported as mean ± standard deviation. Over the first 12 months, patients in the 2.0 mg PRN group (n = 273) required 6.9 ± 2.4 injections compared with 7.7 ± 2.7 injections in the 0.5 mg PRN group (n = 275) (4). This corresponded to a reduction of 0.8 injections with 2.0 mg PRN (p < 0.001; 95% CI: −1.23 to −0.37). By month 24, the gap widened substantially: the 2.0 mg PRN group required 11.9 ± 5.0 injections versus 14.2 ± 5.7 injections in the 0.5 mg PRN group (n = 237 in both groups) (5), representing a reduction of 2.3 injections over two years (p < 0.001; 95% CI: −3.27 to −1.33).

## 3 Discussion

Our reanalysis of the HARBOR trial demonstrates that, over both the first 12 months and the full 24-month period, patients in the 2.0 mg ranibizumab PRN group required significantly fewer injections than those in the 0.5 mg PRN group. Specifically, the higher-dose regimen reduced the injection burden by nearly one injection during the first year and by more than two injections over two years, a clinically meaningful finding. Furthermore, after excluding the initial three loading doses, the 24-month results of the HARBOR trial indicate that the mean treatment interval in the 2.0 mg PRN group was 12.5 weeks compared with 9.9 weeks in the 0.5 mg PRN group, representing an extension of more than 2.6 weeks (5). This extended interval is consistent with the broader trend toward longer dosing intervals observed with newer anti-VEGF therapies.

While the original interpretation of HARBOR trial rightly focused on visual acuity, current clinical priorities often include durability as a key secondary goal. Modern phase 3 trials have set new benchmarks for treatment intervals. For instance, the PULSAR trial of high-dose aflibercept (8.0 mg) reported that more than 70% of patients maintained dosing intervals of either 12 or 16 weeks through week 48 (1), with 31% achieving intervals of up to 20 weeks through 96 weeks of follow-up (6). Similarly, the TENAYA and LUCERNE trials demonstrated that 59% and 66.9% of patients receiving faricimab (6.0 mg) could achieve intervals of 16 weeks through 112 weeks of follow-up (2). Against this backdrop, the 12.5-week mean interval achieved with ranibizumab 2.0 mg in HARBOR trial is highly comparable to these contemporary standards. In the context of modern long-acting anti-VEGF agents, high-dose ranibizumab therefore serves as a historical proof-of-concept demonstrating that increasing the therapeutic load can effectively prolong the duration during which intraocular drug levels remain above the therapeutic threshold. Recognizing this historical consistency further supports the rationale for the dose-escalation strategies currently employed in clinical practice.

From a pharmacokinetic perspective, the estimated human vitreous half-lives of currently available anti-VEGF agents are relatively similar, generally ranging from approximately 7 to 10 days (7, 8). Therefore, differences in half-life alone are unlikely to be the primary determinant of treatment interval. Instead, treatment durability may be more closely related to strategies that increase the initial intraocular molar concentration or introduce synergistic biological mechanisms (9). Faricimab achieves extended durability through dual inhibition of VEGF-A and angiopoietin-2, which contributes to vascular stabilization (8, 10). In the case of ranibizumab, a fourfold increase in dose (from 0.5 mg to 2.0 mg) increases the initial intraocular drug concentration, allowing drug level to remain above the minimum therapeutic threshold required for VEGF suppression for approximately two additional half-lives. This pharmacokinetic effect may translate into an extension of the treatment interval by approximately 2.6 weeks. Notably, the clinical durability of high-dose aflibercept exceeds projections derived strictly from pharmacokinetic half-life extrapolation. While the standard 2.0 mg regimen is typically administered at 8-week intervals, the 8.0 mg formulation has successfully sustained treatment intervals of 12 to 16 weeks in patients with nAMD. This extended efficacy suggests that complex pharmacodynamic mechanisms, rather than simple pharmacokinetic decay, mediate the prolonged therapeutic action of high-dose aflibercept (9). However, it remains undetermined whether a comparable phenomenon extends to high-dose ranibizumab.

Minimizing treatment burden is an important consideration in the management of nAMD, as frequent clinic visits and intravitreal injections place substantial demands on both patients and healthcare systems (11). In real-world practice, where patient adherence is often suboptimal and undertreatment is an important contributor to long-term vision loss (12), saving approximately one injection per year may represent a clinically meaningful reduction in treatment burden. Each injection involves more than the cost of the drug; it requires caregiver time, transportation, and can contribute to anxiety among elderly patients (13). Reducing the number of injections could help alleviate clinic congestion, improve care for acute patients, and lower burnout rates among retina specialists. Therefore, treatment strategies that reduce injection frequency may offer clinically relevant advantages beyond visual acuity outcomes alone.

## 4 Conclusion

The previous assumption that high-dose ranibizumab does not demonstrate a clinically meaningful difference in efficacy or durability warrants reconsideration. Although visual acuity outcomes were comparable, this reanalysis demonstrates that high-dose ranibizumab (2.0 mg) significantly reduces treatment burden compared to the standard dose (0.5 mg) for nAMD. Moreover, the treatment interval achieved with the high-dose ranibizumab appears broadly comparable to the intervals reported with newer anti-VEGF agents. Signals of improved durability with dose escalation were already evident within the HARBOR trial. Viewed from a treatment-burden perspective, these findings align with dosing strategies adopted in more recent high-dose anti-VEGF studies and support the relevance of dose escalation in the development of longer-acting therapies.
